# Preparation of Monodispersed Cs_0.33_WO_3_ Nanocrystals by Mist Chemical Vapor Deposition for Near-Infrared Shielding Application

**DOI:** 10.3390/nano10112295

**Published:** 2020-11-20

**Authors:** Lei Huang, Hua Tang, Youjun Bai, Yong Pu, Lu Li, Jiang Cheng

**Affiliations:** 1 Co-Innovation Center for Micro/Nano Optoelectronic Materials and Devices, Chongqing University of Arts and Sciences, Chongqing 402160, China; winnerhuang@foxmail.com (L.H.); htang44@163.com (H.T.); puyong1216@163.com (Y.P.); lli@cqwu.edu.cn (L.L.); 2 Chongqing Changan Automobile Co., Ltd., Chongqing 400023, China; 3 Department of Engineering and Technology, Hainan College of Economics and Business, Haikou 571127, China; 642baiyoujun@163.com

**Keywords:** cesium tungsten bronze, near-infrared shielding, mist chemical vapor deposition, composite coating

## Abstract

In this study, single-phase Cs_0.33_WO_3_ nanocrystals were synthesized by a novel mist chemical vapor deposition method. As prepared, Cs_0.33_WO_3_ nanocrystals exhibited a microsphere-like appearance constructed with angular crystal grains with an average size of about 30–40 nm. Characterization by X-ray photoelectron spectroscopy indicated that Cs_0.33_WO_3_ nanocrystals consisted of mixed chemical valence states of tungsten ions W^6+^ and W^5+^, inducing many free electrons, which could scatter and absorb near-infrared (NIR) photons by plasmon resonance. These Cs_0.33_WO_3_ microspheres consisted of a loose structure that could be crushed to nanoscale particles and was easily applied for producing long-term stable ink after milling. Herein, a Cs_0.33_WO_3_/polymer composite was successfully fabricated via the ultrasonic spray coating method using mixed Cs_0.33_WO_3_ ink and polyurethane acrylate solution. The composite coatings exhibited excellent IR shielding properties. Remarkably, only 0.9 mg cm^−2^ Cs_0.33_WO_3_ could shield more than 70% of NIR, while still maintaining the visible light transmittance higher than 75%. Actual measurement results indicate that it has really good heat insulation properties and shows good prospect in heat insulation window applications.

## 1. Introduction

In modern buildings, a large part of heat exchange is contributed by heat radiation from the glass windows or glass walls, i.e., a significantly large amount of energy is consumed by the window glass. The development of efficient transparent heat-shielding (THS) glass coating technology is quite necessary for modern energy-saving buildings [1,2,3]. Among the currently used THS materials, cesium-doped tungsten bronzes (Cs_0.33_WO_3_) have attracted significant research interest because of their great heat-shielding ability in near-infrared (NIR) range (780–2500 nm) with high visible transparency [3,4,5,6,7]; e.g., only 1 mg cm^−2^ Cs_0.33_WO_3_ could shield more than 75% of heat radiations. Moreover, owing to its simple composition and good solubility of the elements, it could be synthesized easily via many solution methods or solid-state synthesis methods [8,9,10]. 

Recently, both the synthesis and nanosizing of Cs_0.33_WO_3_ have achieved notable progress, indicating that Cs_0.33_WO_3_-based THS coating could easily be fabricated cost-effectively in the very near future. The solution method including the hydrothermal or solvothermal technique is popular for its simple and facile process with a low reaction temperature [11,12,13,14]. Notably, the synthesis and nanosizing for solution-processed Cs_0.33_WO_3_ usually occur simultaneously, and the morphology and size of the nanocrystals are also easily controllable [15]. If we pursue a high yield facing industrial fabrication, the solid state calcination method is a good choice, although it usually needs high temperature, and it is difficult to control the morphology, size, and agglomeration of particles [1,16]. According to literature, Cs-salts and ammonium tungstate mixed crystals were used as the starting materials and co-precipitation from aqueous solution, the solid reaction could be conducted thoroughly at 800 °C, and the single-phase Cs_0.33_WO_3_ could be prepared successfully [15]. For this approach, the post-milling process with nano-surface modification is crucial to obtain the nanosized final products [17,18].

Significant research efforts have been devoted to the study of the synthesis, characterization, and NIR shielding property of Cs_0.33_WO_3_ nanocrystals. The progress endows researchers with enough encouragement to further investigate the practical application and prepare for industrial production. The study on THS coatings directly cast on glass using paste or inks has provided the preliminary results of NIR shielding and exhibited attractive application prospects [2,19]. Moreover, larger-scale preparation of paraffin wax–Cs_0.33_WO_3_ composite coatings using automatic film applicator provides positive information leading to steps to successful commercialization [20]. Nevertheless, it is believed that a lot more systematic explorations are still needed before its widespread application. A mature recipe of ink, capable of mixing well with conventional solvent and adhesive and containing long-term suspended particles, is still highly desirable. Moreover, a solution is required to apply this THS onto the glass already fixed to the existing buildings in order to easily upgrade the most normal windows to heat-shielding windows. Of course, the premise is that the THS coating should enhance aesthetic appeal and be cost-effective.

Aiming at this requirement, herein, the mass fabrication of Cs_0.33_WO_3_ crystals was improved using the mist chemical vapor deposition (mist-CVD) method, and alcohol-based Cs_0.33_WO_3_ ink was successfully prepared via wet grinding with a silane coupling agent. The ink was obtained in a high yield and exhibited long-term dispersion stability. Importantly, the ink could be well mixed with most of conventional organic solvents and ultraviolet (UV) adhesives. Using a self-designed spray apparatus [21,22] and a UV box, smooth polymer nanocomposite coating was fabricated on glass. The composite coating exhibits a significantly high heat-shielding performance in practical applications. This technology also has a good prospect in up-gradation of the existing building glass after further optimization.

## 2. Materials and Methods

### 2.1. Chemicals

Ammonium metatungstate ((NH_4_)_6_H_2_W_12_O_40_·*x*H_2_O, 99.9%) and cesium hydroxide hydrate (CsOH·H_2_O, 99.95%) were purchased from Macklin Inc. (Shanghai, China). Silane coupling agents were purchased from Shintech (KBM-602, Tokyo, Japan). Polyurethane acrylate (PUA) prepolymers were purchased from Yungu Electronics Technology Co., Ltd. (Dongguan, China).

### 2.2. Preparation of Cs_0.33_WO Nanoparticles and Ink

**Figure 1:**
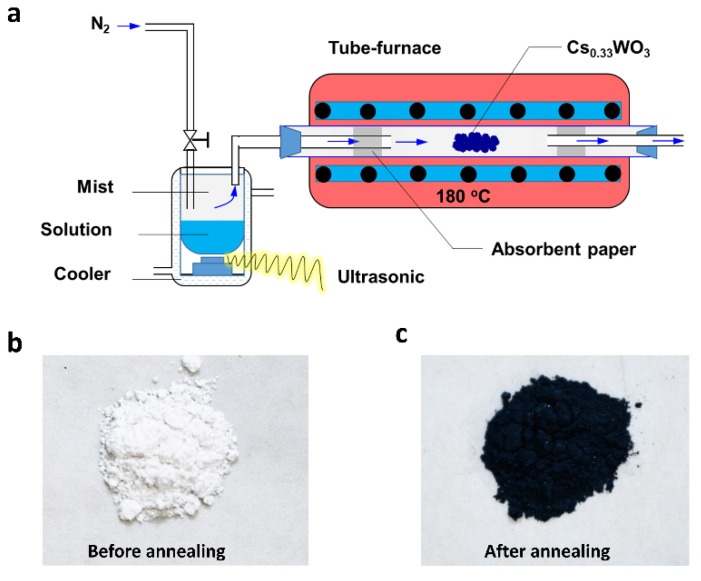
Preparation of Cs_0.33_WO nanoparticles (NPs): (**a**) schematic illustration of a mist chemical vapor deposition (mist-CVD apparatus), and photographs of Cs_0.33_WO NPs (**b**) before and (**c**) after annealing in reducing atmosphere at 550 °C.

The mist-CVD apparatus consists of an ultrasonic transducer, a tube furnace, and pipe fittings, as shown in Figure 1a. The purpose of the ultrasonic transducer is to generate an ultrasonic wave, which can continuously convert the solution into mist. Some absorbent papers were used to prevent the condensation of water vapor on the tube wall. Briefly, Cs_0.33_WO_3_ nanoparticles (NPs) were synthesized using the following procedure: ammonium metatungstate (29.56 g, 10 mmol) and cesium hydroxide hydrate (6.45 g, 38.4 mmol) were thoroughly dissolved in deionized water (200 mL). The solution was then atomized to mist at a rate of 5 mL min^−1^. N_2_ was used as the carrier gas, which brought the mist to the tube furnace. The mist was then dried rapidly and transformed to white powder at a temperature of 180 °C, as shown in Figure 1b. Then, it was transferred to a ceramic boat and heated at 550 °C in a reducing atmosphere containing 95% N_2_ and 5% H_2_ for 3 h. After naturally cooling it down to room temperature, dark blue powder (~8.5 g) was collected, as shown in Figure 1c. Cs_0.33_WO_3_ ink was prepared after wet grinding in a laboratory ball mill for 24 h at a speed of 800 rpm. Before milling, NPs (1.5 g) were mixed with a solution containing methoxy ethanol (30 mL) and KH-602 coupling agent (1.5 mL).

### 2.3. Preparation of Cs_0.33_WO_3_/Polymer Nanocomposites Coatings

Soda–lime glasses were used as substrates for coating fabrication. All the substrates were cleaned with alkaline detergent (RM10-07, Rigorous Co., Ltd., Shenzhen, China) and were consecutively immersed in an ultrasonic bath with de-ionized water before drying with nitrogen flux. Cs_0.33_WO_3_/polymer nanocomposites coating was fabricated utilizing an ultrasonic spray deposition apparatus, which was previously used in our study to prepare large-scale transparent conductive films and photovoltaic devices [21,22]. The spray solution consisted of dilute Cs_0.33_WO_3_ ink and PUA prepolymer. During the coating process, an optimized flow rate for carrier gas (N_2_) and solution spraying rate were held constant at 20 L min^−1^ and 0.15 mL min^−1^, respectively. The wet coating was then pre-dried in vacuum at room temperature for 2 h. Finally, the solid Cs_0.33_WO_3_/polymer nanocomposites coating was formatted in a UV box, in which the PUA in nanocomposites polymerized completely.

### 2.4. Material Characterization and Device Testing

The crystal structure and composition were characterized via X-ray diffraction (XRD, AXS D8 Advance, Bruker, Karlsruhe, Germany) and energy-dispersive X-ray spectroscopy (EDS, Quantax 400, Bruker, Karlsruhe, Germany). The NPs and coating morphologies were characterized via scanning electron microscopy (SEM, Merlin, Zeiss, Oberkochen, Germany). The chemical composition and electronic structure of Cs_0.33_WO_3_ NPs were analyzed using X-ray photoelectron spectroscopy (XPS, ESCALAB 250xi, ThermoFisher, Waltham, USA). The core-level XPS multi-peaks were fitted using the Gauss multi-peak fitting method. During the fitting for W4f7/2 and W4f5/2, the interval between them was fixed as 2.1 eV. Optical transmittance spectra of the coating were obtained using a UV–Vis–IR spectrophotometer (Cary 5000, Agilent, Santa Clara, CA, USA). The thickness of the coatings was measured using a stylus profile meter (Alpha-Step D-100, KLA-Tencor, MI, USA).

## 3. Results and Discussions

**Figure 2:**
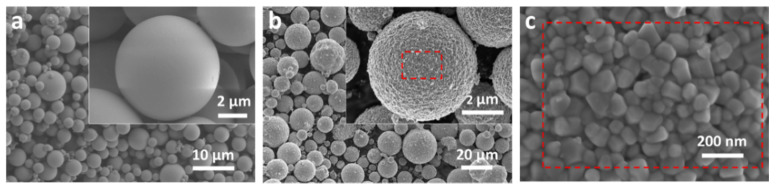
SEM micrographs of Cs_0.33_WO_3_, exhibiting morphology: (**a**) before and (**b**) after annealing, and (**c**) a magnified image for annealed sample.

In the mist-CVD method, the size of droplets of mist was very small (less than 20 μm), the drying and pyrolysis processes were very rapid, and the crystallization was hardly limited by space. Thus, monodispersed Cs_0.33_WO_3_ could be easily precipitated from mist droplets. Figure 2a,b shows the SEM micrographs of mist-CVD prepared Cs_0.33_WO_3_ before and after annealing. Both samples consist of microspheres with different diameters (approximately 2–5 μm). The sample before annealing exhibits a very smooth surface, reflecting its amorphous appearance. By contrast, the surface of annealed microspheres shows a rough appearance. A magnified image shows that the surface of annealed microspheres is constructed with angular crystal grains with an average size of about 30–40 nm.

**Figure 3:**
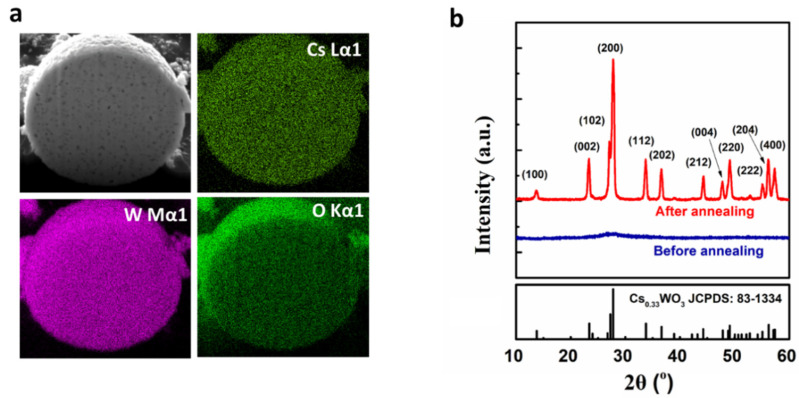
Elemental and structural analysis of Cs_0.33_WO_3_: (**a**) focused ion beam (FIB)-cut cross image and EDS mapping of Cs, W, and O elements and (**b**) XRD patterns of Cs_0.33_WO_3_ before and after annealing.

In order to further study the inner structure of Cs_0.33_WO_3_, one microsphere was cut open via a focused ion beam (FIB). FIB-cut cross shows that the particles are compact solid balls piled up with crystal grains both inside and on the surface. The EDS mapping technique was employed to analyze the components by investigating the element distribution. Figure 3a demonstrates that the Cs, W, O signals are evenly distributed, indicating the presence of homogeneous chemical composition in as-prepared product. The XRD pattern shows the exact crystal structure and composition information. The broad XRD pattern peak at 27.8° corresponding to Cs_0.33_WO_3_ (200) is observed for the sample before annealing, indicating its amorphous structure, which is in good agreement with SEM observation. By contrast, the sample annealed at 550 °C in Ar/H_2_ atmosphere shows good crystallinity, which exhibits hexagonal cesium tungsten oxide structure well matched with the standard PDF (No.83-1334), and no obvious impurity peaks can be observed in the pattern, indicating the as-prepared product consists of single-phase Cs_0.33_WO_3_ nanocrystals.

**Figure 4:**
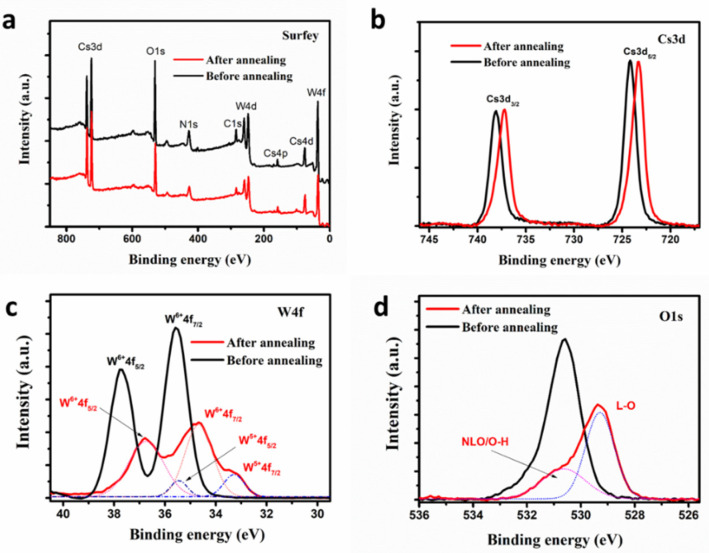
XPS analysis of Cs_0.33_WO_3_ nanocrystals synthesized via mist-CVD: (**a**) The survey scan and the core-level spectra of (**b**) Cs3d, (**c**) W4f, and (**d**) O1s before and after annealing.

XPS spectra of Cs_0.33_WO_3_ nanocrystals before and after annealing in reducing atmosphere were obtained to further access compositional information, and the results are shown in Figure 4a–d. Clearly, there is no obvious difference in the XPS survey (Figure 4a) for samples before and after annealing, indicating no significant change in the composition of Cs_0.33_WO_3_ during the annealing process. However, some variation could be found in core-level spectra. This shows that the main XPS peaks (including Cs3d, W4f, and O1s) of annealed sample monotonously shift to lower binding energies probably due to further pyrolysis and increasing crystallinity [23,24]. Core-level XPS of the unannealed sample shows two spin–orbit doublets, W4f_7/2_ and W4f_5/2_, peaked at 35.8 and 37.9 eV, respectively. After annealing, the core-level XPS spectrum of W4f appears as a multi-peak feature. The curve can be fitted as two groups of spin–orbit doublets with W4f_7/2_ and W4f_5/2_. The interval between them is fixed as 2.1 eV during the fitting. The peaks at 34.7 and 36.8 eV are ascribed to W4f_7/2_ and W4f_5/2_ of W^6+^. Their full widths at half maximum (FWHMs) are 1.29 and 1.67 eV, respectively. The other two peaks at 33.1 and 35.2 eV are attributed to W^5+^ because of the lower Coulomb force. Their FWHMs are 0.99 and 0.82 eV, respectively.

It is well known that the strong NIR shielding ability of cesium tungsten bronze stems from localized surface plasmon resonance induced by a high density of free electrons [25,26]. When it was annealed in reducing gas (H_2_), the solid–gas reaction could be elaborated as the following.

$$x{\;H}_{2}+Cs_{0.33}{\mathrm{WO}}_{3}≜Cs_{0.33}{\mathrm{WO}}_{3-x}+x\;Vö+2x\;e^{\prime }+x{\;H}_{2}O$$

During the annealing process, a small part of the lattice oxygen could be extracted by H_2_, resulting in much oxygen vacancy and bringing a quantity of free electrons. Simultaneously, many W^6+^ in Cs_0.33_WO_3_ would be reduced to W^5+^ because of the loss of lattice oxygen. Thus, the increase of W^5+^ is a substantial piece of evidence, illustrating that many free electrons may be introduced, and they could enhance the NIR shielding property. It was thus suggested that the unannealed samples contained only W^6+^, some of which were reduced to W^5+^ after being annealed in the reduced atmosphere, and simultaneously brought many free electrons, resulting in significant enhancement of NIR shielding [4,6,11]. The O1s XPS spectra exhibit asymmetric line shapes (Figure 4d). The peak with lower binding energy (528.2 eV) and a FWHM of 1.27 eV corresponds to lattice oxygen (LO) in the stoichiometric WO_3_ phase (O atoms binding with W^6+^). The second peak, at 530.8 eV (FWHM ~1.95 eV), may originate from surface O-H states or non-lattice oxygen (NLO) in the nonstoichiometric phase (O atoms binding with W^5+^), which is in agreement with W4f XPS peaks [27,28].

**Figure 5:**
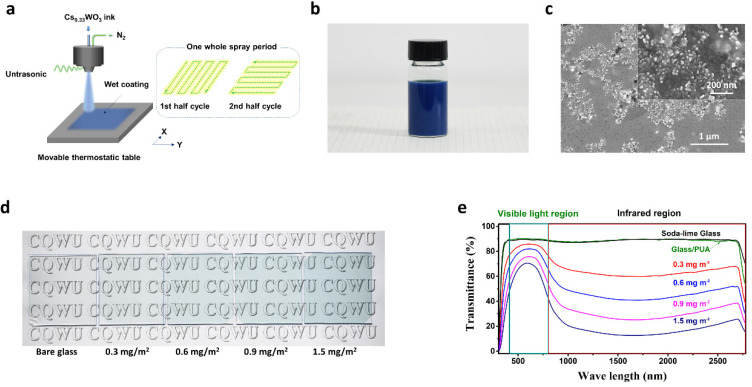
Spray deposition of Cs_0.33_WO_3_/polyurethane acrylate (PUA) composite coatings: (**a**) a sketch for spray deposition, (**b**) Cs_0.33_WO_3_ ink, SEM micrographs of (**c**) dried Cs_0.33_WO_3_ ink, and (**d**) a photograph and (**e**) UV–Vis–IR transmittance of Cs_0.33_WO_3_/PUA composite coating with Cs_0.33_WO_3_ concentration ranging from 0.3–1.5 mg m^−2^.

To realize application in NIR shielding, Cs_0.33_WO_3_/PUA composite coatings were prepared via the ultrasonic spray-coating technology we previously used, and its sketch is shown in Figure 5a. Assisted by the automatic X–Y table, herein, a larger-scale coating with uniform thickness was prepared. For convenience, the Cs_0.33_WO_3_/PUA composite coatings were prepared with a consistent thickness, in which the concentration of Cs_0.33_WO_3_ was varied. The spray solution consisted of PUA prepolymer, ethoxyethanol, and Cs_0.33_WO_3_ ink. The ink was prepared by wet grinding using annealed Cs_0.33_WO_3_ with methoxyethanol and KH-602 coupling agent with the solid content of approximately 5%. Figure 5b shows a photograph of ink, exhibiting very long-term dispersion stability (as long as several months). In order to study the particles in the ink, a drop of ink was deposited on glass and dried for investigation. SEM micrographs (Figure 5c) show that this ink consisted of nanocrystals with an average size of 30–40 nm. This size is consistent with the size of crystals shown in Figure 2c, indicating that the Cs_0.33_WO_3_ microspheres were loose and could be crushed to nanoscale particles after milling. By using the spray solution, Cs_0.33_WO_3_ nanocrystals could be well dispersed in PUA prepolymer after UV curing. In order to study the NIR shielding property, a 5-μm-thick Cs_0.33_WO_3_/PUA composite coating was prepared in a soda–lime glass with the variation in the concentration of Cs_0.33_WO_3_ from 0.3 to 1.5 g m^−2^, and their photographs are shown in Figure 5d. Figure 5e illustrates their transmittance spectra in the range of 300–2800 nm. The difference between soda–lime glass and pure PUA (5-μm-thick) coated glass is very small. Either of them exhibits a high transmittance (~90%) in the visible and NIR regions, showing both glass and PUA have no obvious NIR shielding ability. The 5-μm-thick composite layer with only a small amount of Cs_0.33_WO_3_ (0.3 g/m^2^) on glass shows an obvious NIR shielding property, i.e., it is highly transparent (~83%) in the visible range, while a much lower transmittance (66%) in NIR regions is witnessed. When the concentration of Cs_0.33_WO_3_ was increased from 0.3 to 0.9 mg m^−2^, the visible transmittance decreased to 74% and NIR transmittance sharply decreased to below 30%. When the concentration was increased to 1.5 mg m^−2^, the NIR transmittance was below 17%, while the visible transmittance was still higher than 65%, indicating its great IR shielding property and showing a good application prospect in energy-saving buildings.

**Figure 6:**
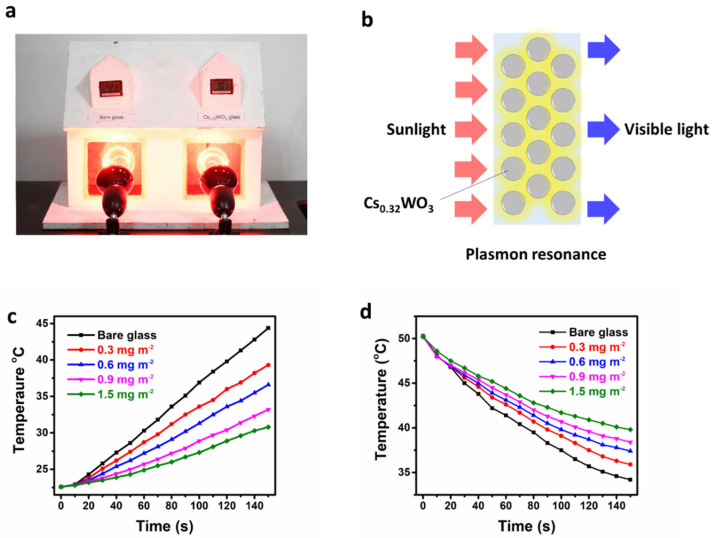
Heat insulation effect of Cs_0.33_WO_3_/PUA composite coatings: (**a**) photograph of a model, (**b**) heat insulation mechanism of Cs_0.33_WO_3_/PUA composite coating, (**c**) heating curve, and (**d**) cooling curve of model Cs_0.33_WO_3_/PUA composite coating with varying Cs_0.33_WO_3_ concentration.

To demonstrate the potential application of Cs_0.33_WO_3_/PUA composite coatings, a homemade model (Figure 6a) including two individual independent rooms that were, respectively, installed with bare glass and Cs_0.33_WO_3_/PUA composite coatings-coated glass was used to carry out the simulation experiment of exposure to irradiation. Cs_0.33_WO_3_ nanocrystals were well dispersed in PUA; therefore, most of the IR photons could be scattered and absorbed after reflection or refraction several times by plasmon resonance of free electrons [11,29,30], as shown in Figure 6b. Figure 6c shows the time–temperature curve for the window using glass with different concentrations of Cs_0.33_WO_3_ radiated by 100 W heat lamps. It was found that the temperature of the room using the Cs_0.33_WO_3_/PUA composite coatings-coated window increased much slower than that using a bare soda–lime glass. When a bare soda–lime glass was used, after 150 s, room temperature reached up to 44.4 °C, while the temperature using 0.3, 0.6, 0.9, and 1.5 mg m^−2^ Cs_0.33_WO_3_ was only 39.3, 36.6, 33.2, and 30.8 °C, respectively, showing its great heat insulation properties. Figure 6d shows the time–temperature curve of cooling to ambient temperature after the room temperature reached 50 °C. The temperature of the room with the Cs_0.33_WO_3_/PUA composite-coated window also decreased more slowly than that using bare glass. After 150 s, room temperature rapidly decreased to 34.2 °C, while the temperature using 0.3, 0.6, 0.9, and 1.5 mg m^−2^ Cs_0.33_WO_3_ remained at 35.9, 37.4, 38.4, and 39.8 °C, respectively, indicating its really good heat insulation properties and good application prospect in heat insulation windows.

## 4. Conclusions

Herein, a single-phase Cs_0.33_WO_3_ was successfully prepared via the novel mist chemical vapor deposition method. Thermal annealing in H_2_ and Ar mixed atmosphere could partly reduce W^6+^ to W^5+^, as well as induce many free electrons, which could scatter and absorb near-infrared photons by plasmon resonance. The Cs_0.33_WO_3_ nanocrystals were easily prepared as long-term stable ink, which was used for the fabrication of Cs_0.33_WO_3_/PUA composite coatings via spray deposition. This composite coating exhibited a near-infrared shielding and thermal insulation performance with highly visible light transmittance. A small amount of Cs_0.33_WO_3_ could shield the most near-infrared, e.g., 0.9 mg cm^−2^ Cs_0.33_WO_3_ could shield more than 70% of near-infrared, while keeping the visible transmittance still higher than 75%. A practical test using a homemade model exhibited its good insulation properties, and this technique offers a good application prospect in large-scale heat insulation windows.

## References

[1] Chao L., Bao L., Wei W., Tegus O. (2019). A review of recent advances in synthesis, characterization and NIR shielding property of nanocrystalline rare-earth hexaborides and tungsten bronzes. Sol. Energy.

[2] Hano N., Takafuji M., Noguchi H., Ihara H. (2019). Monodisperse Surface-Charge-Controlled Black Nanoparticles for Near-Infrared Shielding. ACS Appl. Mater. Interfaces.

[3] Qi S., Xiao X., Lu Y., Huan C., Xu G. (2019). A Facile method to Synthesize Small Size and Superior Crystalline Cs0.33W_O3_ Nanoparticles for Transparent NIR Shielding Coatings. Crystengcomm.

[4] Liu J., Fan C., Shi F., Yu L., Huang X., Hu S., Chen B., Ran S., Liu S. (2016). Fabrication of Cs_0. 32_WO_3_/SiO_2_ aerogel multilayer composite coating for thermal insulation applications. Mater. Lett..

[5] Chen C., Chen D. (2013). Preparation and near-infrared photothermal conversion property of cesium tungsten oxide nanoparticles. Nanoscale Res. Lett..

[6] Okada M., Ono K., Yoshio S., Fukuyama H., Adachi K. (2019). Oxygen vacancies and pseudo Jahn-Teller destabilization in cesium-doped hexagonal tungsten bronzes. J. Am. Ceram. Soc..

[7] Xu Q., Xiao L., Ran J., Tursun R., Zhou G., Deng L., Tang D., Shu Q., Qin J., Lu G. (2018). Cs_0.33_WO_3_ as a high-performance transparent solar radiation shielding material for windows. J. Appl. Phys..

[8] Liu J., Luo J., Shi F., Liu S., Fan C. (2015). Synthesis and characterization of F-doped Cs_0.33_WO_3__−x_F_x_ particles with improved near infrared shielding ability. J. Solid State Chem..

[9] Sato Y., Terauchi M., Adachi K. (2012). High energy-resolution electron energy-loss spectroscopy study on the near-infrared scattering mechanism of Cs_0.33_WO_3_ crystals and nanoparticles. J. Appl. Phys..

[10] Long C.S., Lu H.H., Lii D.F., Huang J.L. (2015). Effects of annealing on near-infrared shielding properties of Cs-doped tungsten oxide thin films deposited by electron beam evaporation. Surf. Coat. Tech..

[11] Shi F., Liu J., Dong X., Xu Q., Luo J., Ma H. (2014). Hydrothermal synthesis of Cs_x_WO_3_ and the effects of N_2_ annealing on its microstructure and heat shielding properties. J. Mater. Sci. Technol..

[12] Adachi K., Ota Y., Tanaka H., Okada M., Oshimura N., Tofuku A. (2013). Chromatic instabilities in cesium-doped tungsten bronze nanoparticles. J. Appl. Phys..

[13] Yao Y., Zhang L., Chen Z., Cao C., Gao Y., Luo H. (2018). Synthesis of Cs_x_WO_3_ nanoparticles and their NIR shielding properties. Ceram. Int..

[14] Liu G., Wang S., Nie Y. (2013). Electrostatic-induced synthesis of tungsten bronze nanostructures with excellent photo-to-thermal conversion behavior. J. Mater. Chem. A.

[15] Liu J., Shi F., Dong X., Liu S., Fan C., Yin S., Sato T. (2015). Morphology and phase controlled synthesis of Cs_x_WO_3_ powders by solvothermal method and their optical properties. Powder Technol..

[16] Takeda H., Adachi K. (2007). Near Infrared Absorption of Tungsten Oxide Nanoparticle Dispersions. J. Am. Ceram. Soc..

[17] Machida K., Adachi K. (2016). Ensemble Inhomogeneity of Dielectric Functions in Cs-Doped Tungsten Oxide Nanoparticles. J. Phys. Chem. C.

[18] Liu J., Xu Q., Shi F., Liu S., Luo J., Bao L., Feng X. (2014). Dispersion of Cs0.33WO3 particles for preparing its coatings with higher near infrared shielding properties. Appl. Surf. Sci..

[19] Xu X., Zhang W., Hu Y., Wang Y., Lu L., Wang S. (2017). Preparation and overall energy performance assessment of wide waveband two-component transparent NIR shielding coatings. Sol. Energy Mat. Sol. Cells.

[20] Zhu Y., Wang B., Zhang Q., Wang H., Zhu J., Liu Y., Zhang Y., Sun X., Zhang X., Yun S. (2019). Paraffin wax–Cs_0.33_WO_3_ composite windows with excellent near infrared shielding and thermal energy storage abilities. Chem. Pap..

[21] Cheng J., Hu R., Wang K., Meng X., Li Y., Yang X., Liao X., Li L., Chong K.B. (2019). Air-Stable Solar Cells with 0.7 V Open-Circuit Voltage Using Selenized Antimony Sulfide Absorbers Prepared by Hydrazine-Free Solution Method. Sol. RRL.

[22] Cheng J., Hu R., Meng X., Li Y., Yan X., Yang X., Liao X., Li L., Pei Q., Chong K.B. (2018). Realization of Large-Scale Polymer Solar Cells Using Ultrasonic Spray Technique Via Solvent Engineering. Sol. RRL.

[23] Dimitrov V., Komatsu T., Sato R. (1999). Polarizability, Optical Basicity and O 1s Binding Energy of Simple Oxides. J. Ceram. Soc. Jpn..

[24] Ishikawa F., Higashi K., Fuyuno S., Morifuji M., Kondow M., Trampert A. (2018). Annealing induced atomic rearrangements on (Ga, In) (N, As) probed by hard X-ray photoelectron spectroscopy and X-ray absorption fine structure. Sci. Rep..

[25] Mattox T.M., Bergerud A., Agrawal A., Milliron D.J. (2014). Influence of shape on the surface plasmon resonance of tungsten bronze nanocrystals. Chem. Mater..

[26] Kang L., Xu W., Wang K., Liang W., Liu X., Gao F., Lan A., Yang Y., Gao Y. (2014). Transparent (NH_4_) _x_WO_3_ colloidal dispersion and solar control foils: Low temperature synthesis, oxygen deficiency regulation and NIR shielding ability. Sol. Energy Mat. Sol. Cells.

[27] Vasilopoulou M., Soultati A., Georgiadou D.G., Stergiopoulos T., Palilis L.C., Kennou S., Stathopoulos N.A., Davazoglou D., Argitis P. (2014). Hydrogenated under-stoichiometric tungsten oxide anode interlayers for efficient and stable organic photovoltaics. J. Mater. Chem. A.

[28] Romanov R.I., Kozodaev M.G., Lebedinskii Y.Y., Perevalov T.V., Slavich A.S., Hwang C.S., Markeev A.M. (2020). Radical-Enhanced Atomic Layer Deposition of a Tungsten Oxide Film with the Tunable Oxygen Vacancy Concentration. J. Phys. Chem. C.

[29] Wang Q., Li C., Xu W., Zhao X., Zhu J., Jiang H., Kang L., Zhao Z. (2017). Effects of Mo-doping on microstructure and near-infrared shielding performance of hydrothermally prepared tungsten bronzes. Appl. Surf. Sci..

[30] Yoshio S., Adachi K., Kubo M. (2019). Cesium desorption mechanism in Cs_0.33_WO_3_ by first-principles molecular dynamics calculations. J. Appl. Phys..

